# Myocardial wall stiffening in a mouse model of persistent truncus arteriosus

**DOI:** 10.1371/journal.pone.0184678

**Published:** 2017-09-29

**Authors:** Christine Miller Buffinton, Alyssa K. Benjamin, Ashley N. Firment, Anne M. Moon

**Affiliations:** 1 Department of Mechanical Engineering, Bucknell University, Lewisburg, Pennsylvania, United States of America; 2 Department of Biology, Bucknell University, Lewisburg, Pennsylvania, United States of America; 3 Department of Molecular and Functional Genomics, Weis Center for Research, Geisinger Medical Center, Danville, Pennsylvania, United States of America; Northwestern University, UNITED STATES

## Abstract

**Background:**

Genetic and epigenetic programs regulate dramatic structural changes during cardiac morphogenesis. Concurrent biomechanical forces within the heart created by blood flow and pressure in turn drive downstream cellular, molecular and genetic responses. Thus, a genetic-morphogenetic-biomechanical feedback loop is continually operating to regulate heart development. During the evolution of a congenital heart defect, concomitant abnormalities in blood flow, hemodynamics, and patterns of mechanical loading would be predicted to change the output of this feedback loop, impacting not only the ultimate morphology of the defect, but potentially altering tissue-level biomechanical properties of structures that appear structurally normal.

**Aim:**

The goal of this study was to determine if abnormal hemodynamics present during outflow tract formation and remodeling in a genetically engineered mouse model of persistent truncus arteriosus (PTA) causes tissue-level biomechanical abnormalities.

**Methods:**

The passive stiffness of surface locations on the left ventricle (LV), right ventricle (RV), and outflow tract (OFT) was measured with a pipette aspiration technique in *Fgf8;Isl1Cre* conditional mutant embryonic mouse hearts and controls. Control and mutant experimental results were compared by a strain energy metric based on the measured relationship between pressure and aspirated height, and also used as target behavior for finite element models of the ventricles. Model geometry was determined from 3D reconstructions of whole-mount, confocal-imaged hearts. The stress-strain relationship of the model was adjusted to achieve an optimal match between model and experimental behavior.

**Results and conclusion:**

Although the OFT is the most severely affected structure in *Fgf8;Isl1Cre* hearts, its passive stiffness was the same as in control hearts. In contrast, both the LV and RV showed markedly increased passive stiffness, doubling in LVs and quadrupling in RVs of mutant hearts. These differences are not attributable to differences in ventricular volume, wall thickness, or trabecular density. Excellent agreement was obtained between the model and experimental results. Overall our findings show that hearts developing PTA have early changes in ventricular tissue biomechanics relevant to cardiac function and ongoing development.

## Introduction

Normal vertebrate cardiac development requires precise integration of genetic, molecular, and mechanical signaling events throughout its complex morphogenesis. Disruption of these complex events that permit normal cardiac development results in congenital heart malformations, the most common type of birth defect (>1% of live births). Many genes regulate the molecular pathways critical for normal cardiac development. Disruption of their function frequently causes defects in the outflow tract such as membranous ventricular septal defect (VSD), double outlet right ventricle, transposition of the great arteries, and persistent truncus arteriosus (PTA). In humans, many of these defects are lethal if not surgically repaired.

In multiple organisms, it has been shown that blood flow and other hemodynamic forces interact with local mechanical properties of developing cardiovascular tissues to engender responses at the genetic, molecular, and cellular levels that direct subsequent morphogenesis [1–5] (see [6] for a review). Early studies in the chick showed that interventions that alter hemodynamic pressure and flow within the developing heart [7–19] (see [20] for a review) have profound consequences for subsequent morphogenesis and ultimate cardiac structure and function.

Genetic systems that reliably create specific cardiovascular defects are an important step towards ultimately dissecting how structural and hemodynamic inputs interact with, and modify, the genetic and molecular programs that control cardiac morphogenesis in normal and pathologic conditions. We have previously shown that conditional deletion of *Fgf8* using the *Isl1Cre* [21] driver generates PTA with 100% penetrance [22]. PTA is a severe cyanotic congenital heart defect that results from failed septation of the conotruncus into right and left ventricular outflow tracts: a large arterial trunk originating from the base of the heart is the outlet for both the ventricles. A large VSD is usually present just below the truncus and the mix of oxygenated and deoxygenated blood from both ventricles is distributed to the coronary, pulmonary and systemic circulations largely based on the vascular resistance of each bed. The truncal valve is frequently incompetent; this has important implications for function and post-repair survival in human patients. Surgical repair involves separating the pulmonary artery (or arteries) from the truncus, and providing egress of deoxygenated blood from the RV to the pulmonary circulation by placing a valved conduit from the RV to the pulmonary artery. Repair of the VSD sends the left ventricular output to the truncus (new aorta), which is also patched at the site of separation of the pulmonary artery [23].

This study questioned whether abnormal blood flow and hemodynamics present during PTA formation cause tissue-level abnormalities. We examined passive tissue mechanical properties, because they have been shown to depend on hemodynamic loads in the developing heart [15,16], are critical to normal cardiac function, and because post-natal structural repair of the defect would miss problems with tissue-level biomechanical properties of structures that appear structurally normal. The animal models were embryonic day (ED) 12.5 control and *Fgf8;Isl1Cre* mutant mouse hearts. Characterization of the mechanical properties of biological materials is often complicated by small volume, irregular geometry, fragility, complicated material behavior, and environmental sensitivity. The pipette aspiration (PA) technique, although still novel, has seen increasing use over the last decade since it can successfully deal with many of these problems. PA involves contacting the tissue with the flat end of a small pipette to create a watertight seal. Vacuum pressure is applied within the pipette to aspirate a small amount of tissue; the relationship between pressure and tissue height in the pipette provides information about the tissue mechanical properties. The technique was recently validated on soft polymeric shams with elastic moduli of 10–370 kPa through comparison with nanoindentation, uniaxial tension, and uniaxial compression [24], and was therefore chosen for this study to measure passive tissue stiffness.

Extracting basic material properties from the relationship between pressure and aspirated height measured in PA requires analysis of a mathematical model. In the case of thin, layered substrates, such as the embryonic heart wall tested here, computational numerical analysis of the models, usually with finite element methods, is required [25–28]. Geometry must be specified and a constitutive, or stress-strain, relationship for the component material(s) assumed. Then the relevant equations of continuum mechanics (e.g., force equilibrium, strain-displacement, stress-strain) are solved numerically over the geometric domain subject to prescribed force and displacement boundary conditions.

Whole-mount confocal imaging was used to obtain geometrical measurements for the mathematical models. We employed tissue autofluorescence, enhanced with paraformaldehyde processing, to provide adequate signal for structural resolution, as has previously been shown for stage HH29 chick heart [29,30]. Because mouse ventricles are heavily trabeculated at ED12.5, serial confocal sections of 2–3 μm thickness are required to adequately resolve the trabecular geometry. BABB clearing was first shown adequate for whole-mount confocal imaging of mouse brains [31] and hearts [32]. Dehydration is necessary and therefore shrinkage is an undesired effect, but it has been quantified in ED12.5 mouse hearts [33].

In this study, passive stiffness at locations on the epicardial surface of the left ventricle, right ventricle, and outflow tract was measured with PA. The results were compared in terms of a strain energy metric based on the measured load-deflection relationship between pressure and aspirated height, and also used as target behavior for finite element models of the left and right ventricles. The hyperelastic material properties of the model were optimized to match the calculated pressure versus aspirated height curve to the experimental results. Both the strain energy metric and calculated model material parameters showed a doubling of stiffness in the left ventricle and quadrupling in the right ventricle in mutants compared to control, while stiffness in the outflow tract itself was unchanged despite its severely abnormal morphology.

## Materials and methods

### Specimen preparation

**Fig 1:**
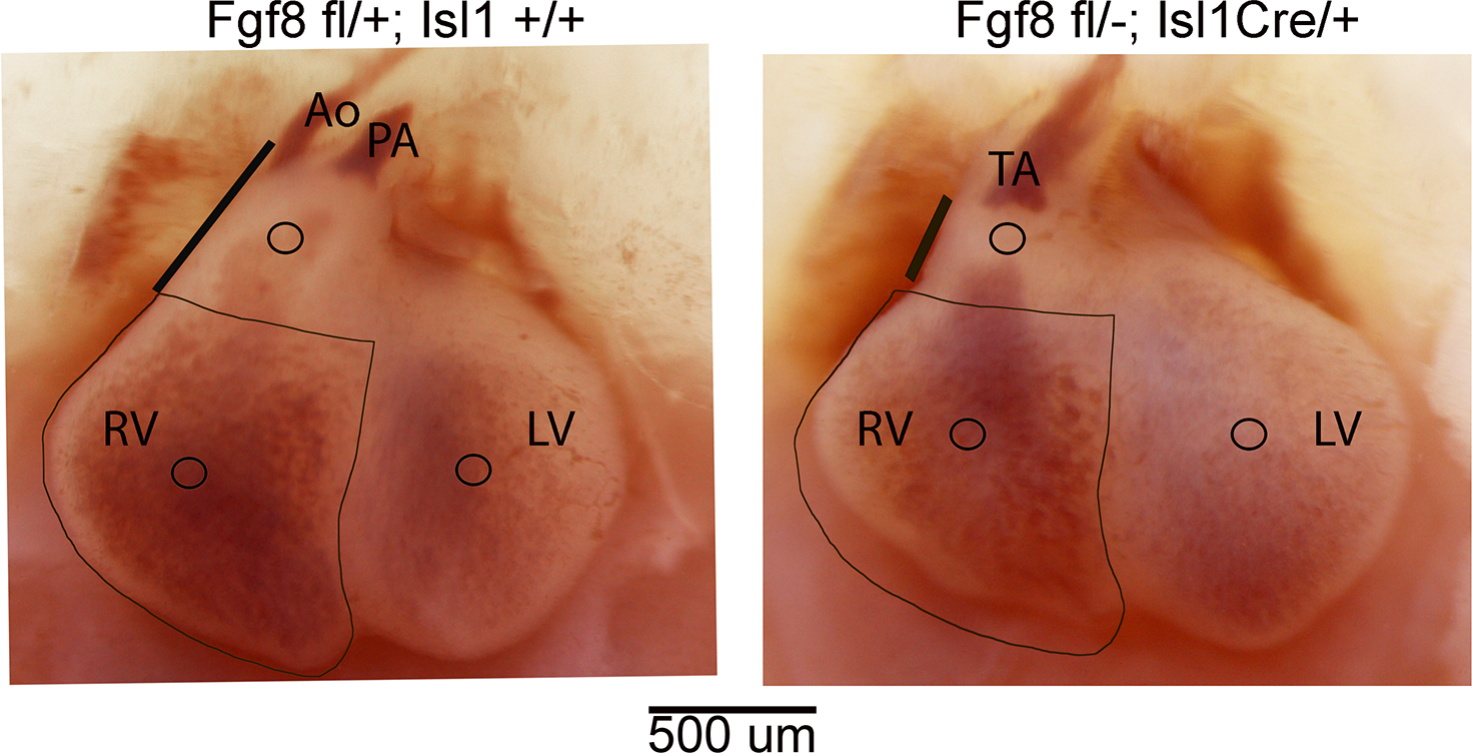
Whole-mount preparations of control and mutant hearts viewed from their ventral surface. Ao, aorta; PA, pulmonary artery; RV, right ventricle; LV, left ventricle; TA, truncus arteriosus. Circles indicate the approximate location of pipette aspiration assay for each structure. Black outlines of the RV are the same size in control and mutant and show the approximate 25% decrease in RV size in mutants. Thick black lines adjacent to the OFT highlight the marked shortening of the mutant OFT.

All animals were maintained on a mixed genetic background. *Fgf8* ^*fl/fl*^ females were mated with *Fgf8*^*+/-*^*;Isl1*^*Cre*/+^ males to generate *Fgf8* conditional mutants (*Fgf8*^*fl/-*^*;Isl1*^*Cre/+*^) and littermate controls (*Fgf8*^*fl/+*^). The *Fgf8;Isl1Cre* mouse mutants are described in detail in Park *et al*. [22] and representative control and mutant hearts at ED12.5 are shown in Fig 1.

All animal protocols were carried out in strict accordance with the recommendations in the Guide for the Care and Use of Laboratory Animals of the National Institutes of Health. The protocol was approved by the Weis Center for Research/Geisinger Clinic IACUC, protocol number 203–17. Maternal euthanasia was achieved with cervical dislocation and diaphragm incision as per the 2013 AVMA Guidelines on Euthanasia: Recommended and Acceptable with Conditions. After maternal euthanasia, ED12.5 mouse embryos were removed into 1x PBS on ice. Hearts were dissected from the embryos, preserving the atria, ventricles, and outflow tract. Yolk sacs were used for genotyping.

For imaging studies, dissected hearts were incubated at room temperature for 20 minutes with rocking in 4% verapamil (in 1x PBS) to arrest the hearts in diastole. They were then washed twice with 1x PBS and immersed in a red blood cell (RBC) lysis solution (155mM ammonium chloride, 10mM Tris HCL, pH7.5) at room temperature for 1 hr. Post RBC-lysis, hearts were fixed with 4% paraformaldehyde for 24–72 hr to increase cardiac tissue autofluorescence, followed by washing twice in 1x PBS, 20 minutes each. Fixed hearts were dehydrated in an ascending series of EtOH (35%, 50%, 70%, 95%), 2 X 30 minutes in each solution, followed by one hr in 100% EtOH. Hearts were cleared in 2:1 benzyl alcohol: benzyl benzoate (BABB) with two changes of BABB with gentle continuous rocking for 30 minutes each. Once cleared, the hearts were stored in BABB in microcentrifuge tubes at 4°C until imaging.

Hearts for pipette aspiration (PA) were prepared as described above through the RBC lysis step. Following RBC lysis, hearts were incubated in 1x PBS at 4° C for one hr twice and then stored at 4° C in PBS until assayed.

### Pipette aspiration

#### Pipette preparation

Borosilicate glass pipettes (0.100 mm ID, 0.170 mm OD, and 100 mm length; VitroCom Technical Glass, Mountain Lakes, NJ) were shortened to 35–50 mm length. The tips were smoothed and flattened by grinding stone and the pipettes glued with cyanoacrylate adhesive to the inside wall of larger pipettes (2.159 mm ID, 2.769 mm OD; Wiretrol II, Drummond Scientific, Broomall, PA) with approximately 25 mm protruding. Hot glue filled the remaining opening of the larger pipettes for an airtight seal. Tubing (2.4 mm ID, 4.0 mm OD; Tygon S3 E-3603) was pressed over the end of the Wiretrol II pipette and connected by T-connector to a pressure transducer (Honeywell SSCDRRM005PDAA5) and syringe.

#### Testing

**Fig 2:**
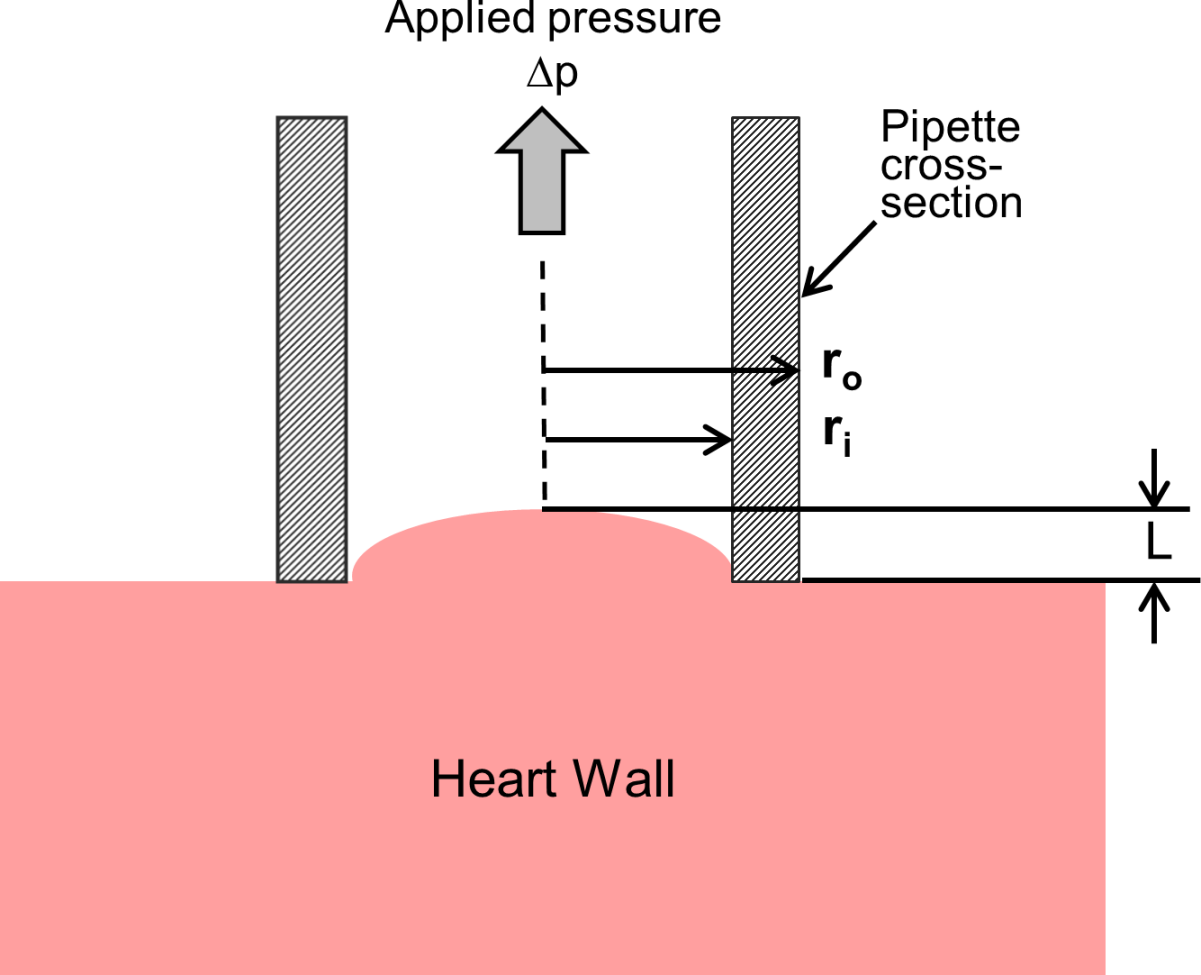
Schematic of an aspiration test shown as a section through the center of the pipette. The tissue is aspirated to a length *L* at the pipette centerline. Here *r*_*i*_ is pipette inner radius, *r*_*o*_ is pipette outer radius, and *Δp* is the aspiration pressure.

Under stereomicroscope viewing, hearts were placed ventral-side-up in a glass specimen chamber, which was then placed on a micromanipulator stage in the experimental setup as previously described [24]. The pipette and heart were aligned in view of an Olympus SZH10 stereomicroscope with the pipette perpendicular to the desired heart surface. As the pipette was lowered toward the heart surface, natural adhesion between the tissue and the glass formed an airtight seal. The test consisted of vacuum pressure applied to the pipette at a continual rate of 0.4 kPa/s, which aspirated tissue into the pipette (Fig 2). A digital camera (DCC1240M-GL, Thorlabs) mounted on the stereomicroscope captured images of the pipette tip and surrounding tissue at 140x magnification while vacuum pressure was applied. The data was recorded by a custom MATLAB program that simultaneously captured the voltage output from the pressure transducer and an image from the camera at a rate of 15 Hz. Three areas were sequentially tested on each heart: left ventricle (LV), right ventricle (RV), and outflow tract (OFT), at the locations shown (Fig 1).

### Analysis

The center deflection *L* (Fig 2) was determined from recorded video with MaxTRAQ software (Innovisions Systems Inc., Columbiaville, MI) using the pipette OD as a caliper basis. Normalized aspirated length $\bar{L}$ was defined as *L/r*_*i*_, where *r*_*i*_ is the inner radius of the pipette. Plotting aspiration pressure *Δp* versus $\bar{L}$ created load-deflection curves, each of which was fit with both a linear and a quadratic trend line for $\bar{L}$ from 0–0.4. The area under the curve in kJ/m^3^ was calculated for two ranges of strain, $\bar{L}$ = 0–0.2 and 0–0.4, by summing individual areas defined between each pair of experimental data points using a trapezoidal method. This area is a measure of the energy per unit volume, or equivalently strain energy density (SED), stored as a result of the deformation by the applied pressure. ANOVA with Tukey post-hoc test determined the statistical significance of differences in strain energy density among the three tested regions, LV, RV, and OFT, in each range for both control and mutant groups. Student’s t-test determined the significance of differences in SED between control and mutant groups at each location and strain range.

### Confocal imaging and reconstruction

#### Microscopy preparation

Microscope slides (25 x 75 x 1.5875 mm) were custom-manufactured of acetal plastic, which is inert to BABB. A glass coverslip (#1.5H high performance, 22 x 22 x 0.170 mm, Schott Nexterion) formed the bottom of a specimen well when mounted with clear silicone gel under a hole (4.76-mm diameter) drilled through the center of the slide.

The contents of the microcentrifuge tubes (hearts and liquid) were transferred with a custom glass suction pipette to three glass block wells, where the heart were identified under epifluorescent illumination (Nikon SMZ 1500). Suction pipettes and wells were coated with 0.1% Triton X-100 to eliminate tissue adhesion. Hearts were then transferred by pipette to the slide chamber, covered in BABB, and gently positioned with dorsal side downward.

#### Confocal microscopic imaging

Confocal imaging was done on a Leica TCS SP5 II inverted confocal microscope with LAS AF (Leica Application Suite) software. A dry 10X objective was used with a numerical aperture of 0.4 and a working distance of 2.2 mm. Relevant laser parameters were: Argon laser at 20% power, visible laser lines at maximum combined blue excitation of 458, 476, and 488 nm and 45% power, PMT2 activated with the Leica/ALEXA488 option and set to grayscale, and detection wavelength from 510 to 570 nm.

Imaging proceeded with an 8-bit, 512 x 512 pixel format at 100 Hz scanning speed and a line average of 8. Gain and offset values were adjusted accordingly for each specimen to allow for maximum signal intensity and contrast in the image. Z-stack parameters were determined with the Z-Wide function to obtain 250–300 steps of 1.7–2.5 μm each in depth, depending on the individual heart dimensions. Scans ran for 3–5 hours to capture the assigned z-stack. The resulting data set was exported and saved as TIFF files with an overlay channels option.

#### Three-dimensional reconstruction and measurement

Images were imported into Amira 6.0 as sequential TIFF files through the Channel 1 conversion option. Pixel dimensions in the X, Y and Z directions were based on data from the Leica MetaData file and compensated for 20% isotropic shrinkage from BABB [33] and refractile foreshortening along the z-axis of 19% [29]. Application of a contrast-limited adaptive histogram equalization filter (CLAHE) compensated for slight variations in signal intensity throughout the data set. The thickness of the compact and trabecular zones in the regions tested by pipette aspiration was measured with the 3D length measuring tool. To determine the density of the trabeculated layer, images from the Amira reconstructions were transferred to ImageMagick (ImageMagick Studio LLC, Landenberg, PA), and the extent of solid tissue as a percentage of total volume occupied was calculated.

For volume calculation, automatic thresholding by grayscale value separated tissue from background, followed by island removal and label smoothing (Gaussian filtering). Further segmentation of the left and right ventricles was done by manual tracing of the chambers with pen tablet on each image, omitting any portions of the developing valves, atria, or outflow tract, and dividing the ventricular septum between the chambers.

### Model

**Fig 3:**
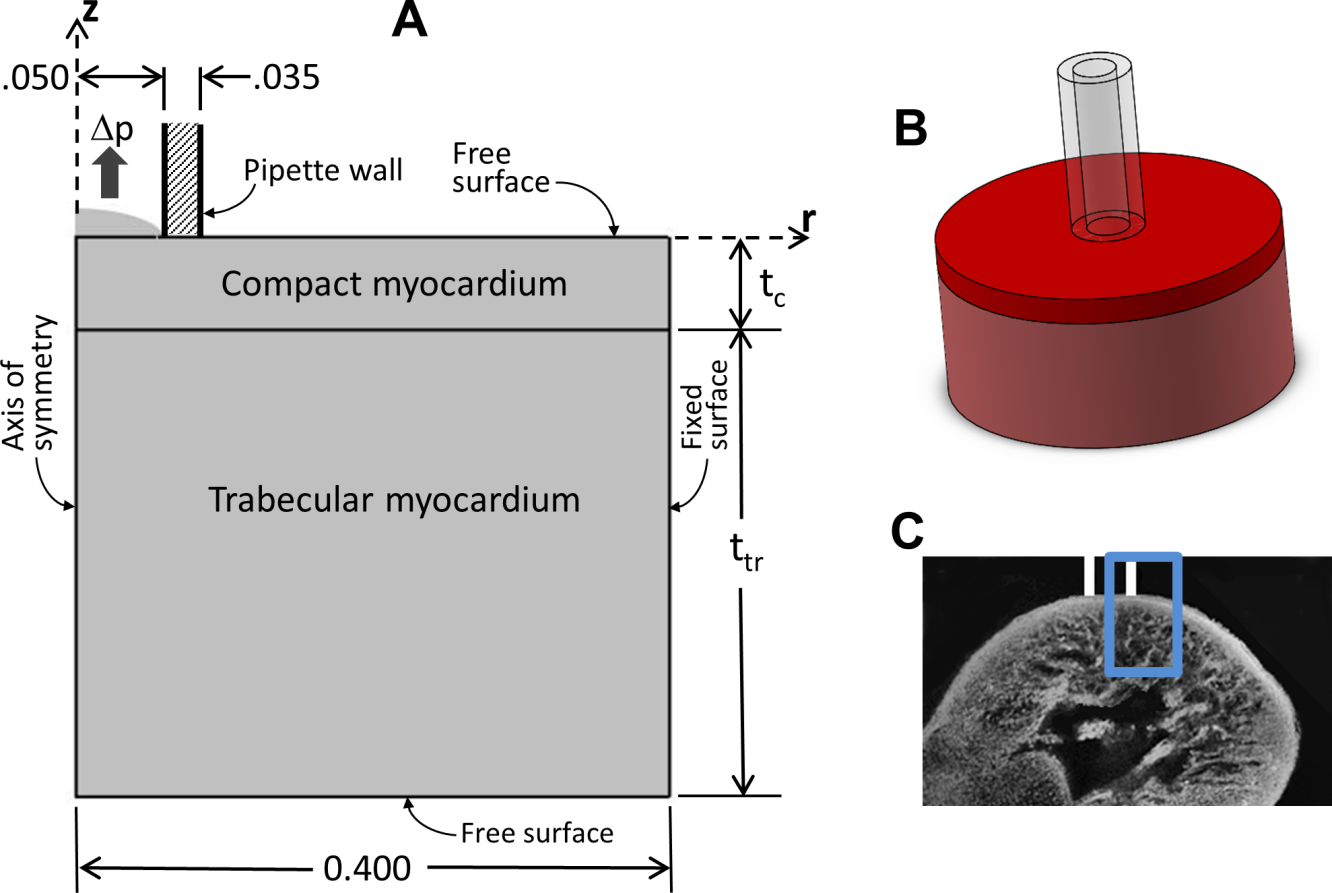
Finite element mathematical model of pipette aspiration test. (A) Two-dimensional section of the geometric model showing the dimensions and the boundary conditions used in the finite element analysis. The model is constructed with a polar coordinate system, with the *z* axis vertical, the *r* axis radial, and the θaxis circumferential (θ is out of the plane of the section and not shown). The model is axisymmetric so no quantities vary with the circumferential coordinate θ. Thicknesses t_c_ represents the compact layer and t_tr_ the trabecular layer. Aspiration pressure Δp is applied uniformly to the tissue surface within the pipette. The outer edge is fixed to ensure a well-posed problem, but as perturbations from the applied pressure dissipate far before this boundary, it has no effect on the solution. All dimensions are in mm. (B) Three-dimensional view of the model, showing the pipette contacting a cylinder of myocardial tissue. The upper, darker red region represents compact zone, while the lower, lighter red region represents trabecular zone. (C) Schematic two-dimensional representation of the pipette contacting the ventricular wall. The region outlined in blue is the approximate region modeled in (A).

Obtaining basic myocardial material properties from the PA test results requires modeling of the domain geometry, loading conditions, and boundary conditions, along with an assumed, parameterized constitutive model for the stress-strain relationship. We created finite element computational models of the PA tests on the LV and RV walls with the ventricular wall tissue represented by a bilayer consisting of compact myocardium on the top, contacting the pipette, perfectly bonded to a layer of trabeculated myocardium underneath. Each layer was assigned a thickness measured from the confocal microscopic reconstructions according to region (LV or RV) and treatment group (control or mutant) (Fig 3A). The region geometry was a cylinder of 0.4-mm radius centered at the pipette centerline (Fig 3B). Four geometric models were thus created: control LV, mutant LV, control RV, and mutant RV (Fig 3B and 3C).

The pipette, with inner radius of 0.050 mm, wall thickness of 0.035 mm, and 0.005-mm circular fillet on both inner and outer wall edges, was assumed to be rigid and fixed with a flat bottom edge contacting the top surface of the compact myocardial layer. A roller boundary condition incorporated traction-free sliding between the myocardial surface and pipette. The outer edge of the cylinder representing ventricular wall was fixed in all directions.

The ventricular tissue was represented as isotropic and homogeneous with a nearly-incompressible, neo-Hookean constitutive relation defined by the Lamé parameter μ and the initial bulk modulus κ. In a neo-Hookean model, these can both be related to the small-strain tangent elastic modulus *E* and Poisson’s ratio ν by the equations

$$\mu =\frac{E}{2(1+\nu )}$$

and

$$\kappa =\frac{E}{3(1-2\nu )}.$$

The modulus *E* was assumed to be the same for the compact and trabecular regions within each of the four geometries. A Poisson’s ratio ν of 0.49 incorporated the nearly-incompressible condition due to the high water content of the tissues. The material parameters of the stress-strain relationship for the trabeculated regions were multiplied by the solid tissue density percentage determined from the analysis of the confocal images. We also analyzed models of each of the four geometries with linear elastic properties to compare with the hyperelastic models, and models without the trabecular zone porosity.

Axisymmetric analysis was implemented in COMSOL Multiphysics software (v5.0, Stockholm, Sweden). The region was meshed with approximately 1200 quadratic tetrahedral elements with a mixed formulation, i.e., pressure added as an independent degree of freedom, to account for the large Poisson’s ratio of 0.49. The use of tetrahedral elements facilitated mesh refinement in the area of largest stress near the contact of the pipette wall with the ventricle. Mesh convergence studies were performed to guarantee the quality of the final solution. The experimental aspiration pressure was applied as a uniform pressure with outward normal on the material surface inside the pipette and incremented from 0 to 3.5 kPa in 0.2-kPa steps to obtain the relationship between aspiration pressure and upward deflection in the aspirated bubble.

To obtain the apparent material parameters of the dual-layer myocardium, the mean experimentally measured load-deflection curves for each group (LV and RV, control and mutant) were used as a target and the modulus *E* for the neo-Hookean constitutive models was iteratively adjusted until the finite element load-deflection curve for the four models best matched the mean experimental curve in a least-squares sense.

## Results

### Pipette aspiration measurements reveal increased passive tissue stiffness in mutant ventricles

**Fig 4:**
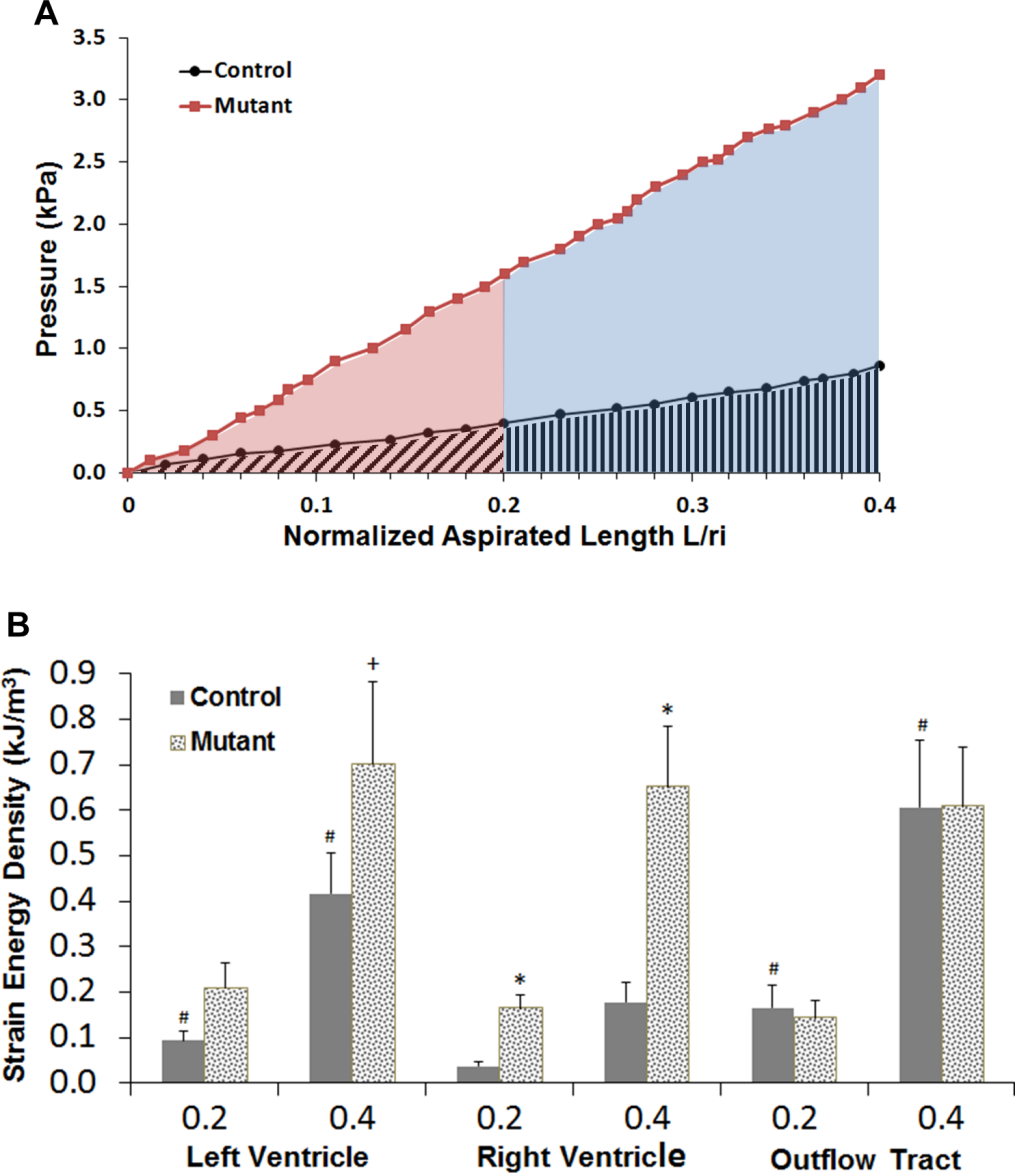
Results of pipette aspiration testing. (A) Experimental measurements from two pipette aspiration tests at the right ventricular location showing how strain energy density was calculated. The hearts from both tests are from the same litter. For the control heart, the area with diagonal cross-hatch represents the strain energy density (SED) calculated for $\bar{L}$ = *L/r*_*i*_ = 0.2; the strain energy density at $\bar{L}$ = 0.4 is the sum of the diagonal cross-hatch and vertical cross-hatch areas. For the mutant heart, the shaded red area represents the SED calculated for $\bar{L}$ = *L/r*_*i*_ = 0.2; the strain energy density at $\bar{L}$ = 0.4 is the sum of the red and blue shaded areas. (B) Mutant hearts developing persistent truncus arteriosus (PTA) had significantly stiffer left and right ventricular walls as measured by pipette aspiration. Mean SED for ED12.5 mouse is shown for ranges of L/r_i_ = 0–0.2 and 0–0.4 (n = 10). Error bars: standard error. Symbols: * on the mutant SED indicates p<0.05 difference from the corresponding control, + indicates p<0.10 difference; # indicates p<0.05 difference between either control OFT or LV location and the RV location.

The load-deflection curves of *Δp* vs $\bar{L}$ from the three regions tested, LV, RV, and OFT, showed increasing aspiration height with pressure (Fig 4A). The aspirated bubble appeared solid in all cases with no separation of tissue layers. The experimental data were described well by a linear equation, with mean R^2^ values from a linear least-squares regression of 0.96 in control and 0.97 in mutant. A quadratic fit to the data raised the mean R^2^ across all locations by only 0.7%.

Comparison of stiffness between control and mutant hearts using the SED metric supported the major hypothesis of this study: in both the LV and RV locations, mutant hearts were significantly stiffer than control (Table 1, Fig 4B). The increase was most pronounced in the RV, where SED, a measure of stiffness, was 4.5 (p = 0.002) and 3.7 (p = 0.003) times larger than in control (0.2 and 0.4 ranges, respectively). In the LV, SED in mutant hearts was 2.2 (p = 0.043) and 1.7 (p = 0.096) times larger than in control. There were no significant differences in SED between control and mutant at the OFT location.

**Table 1:** Measured strain energy density.

	$\bar{L}$ = 0–0.2		${\bar{L}}_{i}$ = 0–0.4	
	Control	Mutant	Control	Mutant
Left Ventricle	0.093 (.021)	0.208 (.057)	0.416 (.091)	0.701 (.183)
Right Ventricle	0.037 (.010)	0.163 (.032)	0.177 (.044)	0.651 (.135)
OFT	0.166 (.050)	0.143 (.038)	0.607 (.147)	0.606 (.132)

Strain energy density in kJ/m^3^ for three testing locations and two ranges of strain as measured by pipette aspiration. Data shown as mean (standard error). $\bar{L}$ is the normalized aspirated length, equal to aspirated length divided by pipette inner radius.

A comparison of PA measurements in control hearts among the three tested locations showed that the OFT was stiffest and the RV was least stiff (Table 1, Fig 4B). The differences were statistically significant (p<0.05) between the RV and OFT and between the RV and LV in both ranges.

### Confocal microscopy and reconstruction provide wall thicknesses, show 25% smaller volume and increased trabecular density in mutant RV

**Fig 5:**
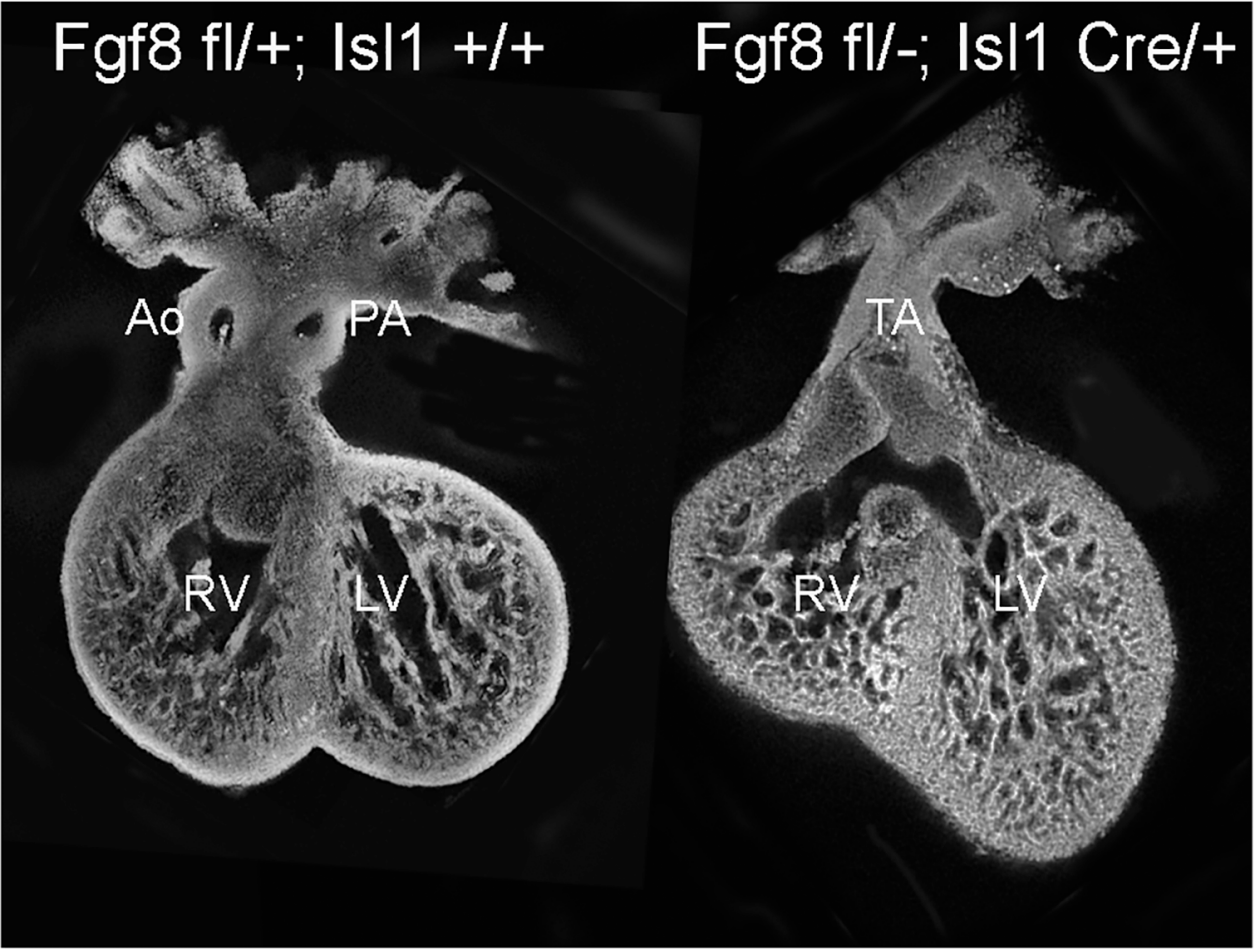
Confocal sections using tissue autofluorescence through comparable planes of (left) control heart and (right) mutant heart.

The BABB clearing method and use of tissue autofluorescence for imaging allowed full laser penetration and capture through the dorsoventral extent of the ED 12.5 mouse hearts and subsequent 3D reconstruction (Fig 5).

Compact layer thickness decreased in both the LV and RV in the mutant hearts, by 27% in the LV but less than half as much, only 12%, in the RV (Table 2). Trabecular density increased by 39% in the mutant RV, but increased only slightly in the mutant LV (Table 2). The mean calculated RV volume in mutant hearts was 25% smaller than in control, 0.139 (.035) vs 0.186 (.028) mm^3^, consistent with whole-mount images (Fig 1). In contrast, the mean calculated LV volume in mutant hearts, 0.176 (.027) (standard error) was very similar to that in control, 0.166 (.014) mm^3^.

**Table 2:** Ventricular measurements from confocal microscopy.

Myocardial Measurement	Control		*Fgf8*^*fl/-*^*;Isl1*^*Cre/+*^ mutants	
	LV	RV	LV	RV
Compact layer (mm)	.062 (.006)	.066 (.008)	.047 (.007)	.057 (.004)
Trabecular layer (mm)	.314 (.009)	.289 (.022)	.307 (.057)	.274 (.004)
Trabecular density (%)	48.2 (2.0)	40.5 (4.6)	51.3 (7.4)	56.2 (1.5)

Mean compact layer thickness, trabecular layer thickness, and trabecular tissue density from the regions tested by pipette aspiration. Measurements were obtained from 3D reconstructions of confocal images and are adjusted for tissue shrinkage from BABB clearing.

### Finite element models show good match with experimental results and doubling and quadrupling of elastic modulus in LV and RV, respectively

When aspiration pressure was mathematically applied to the four models, the finite element analysis showed a bubble of material aspirated into the pipette whose height increased with increasing aspiration pressure. Each model was analyzed with two types of stress-strain relationships, linear and neo-Hookean, by sweeping through a range of material parameters and aspiration pressures. This allowed determination of the basic material parameters that caused each model’s behavior to match the mean experimental load-deflection curves.

**Fig 6:**
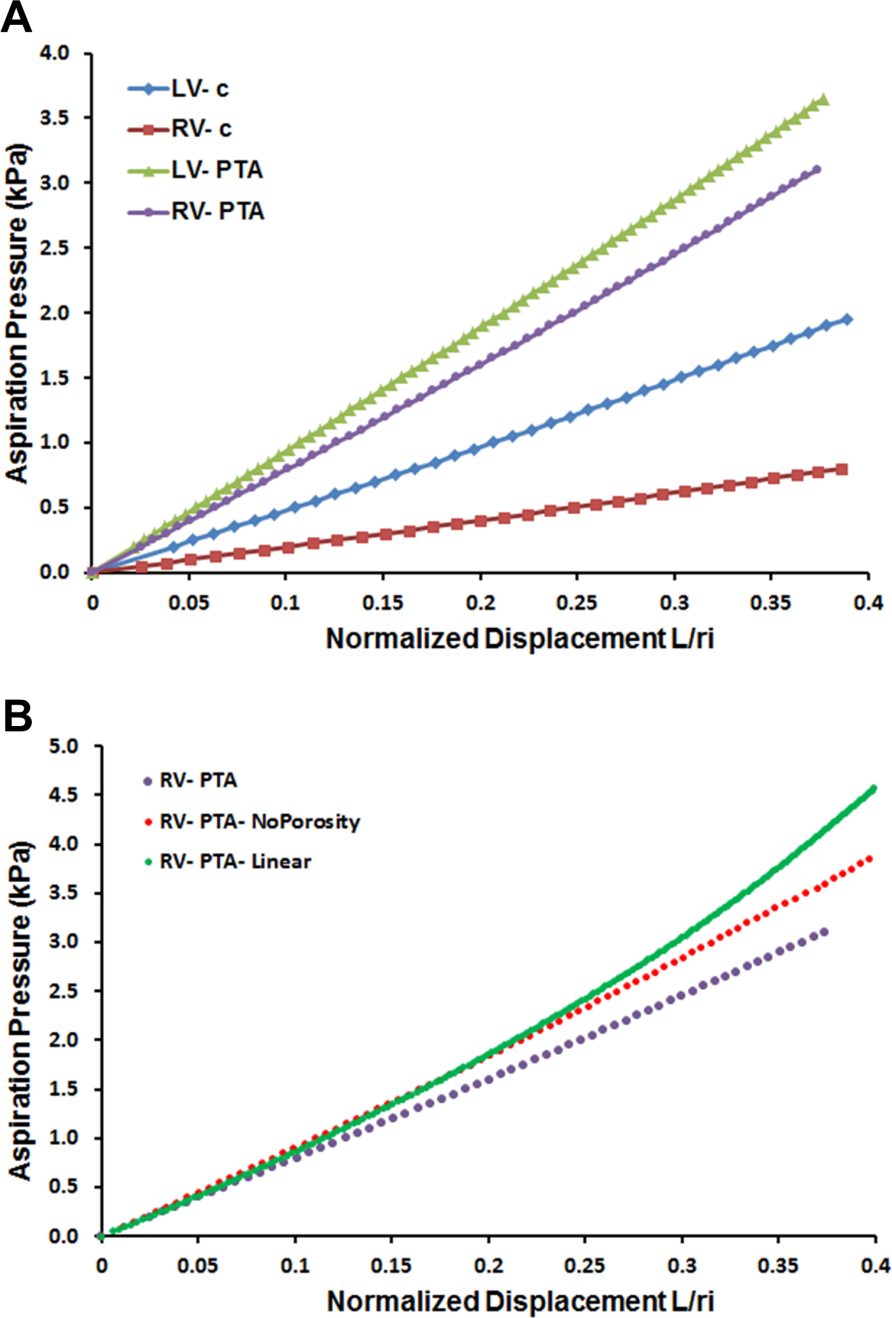
Load-Deflection results from finite element analysis of the models. (A) The relationship between aspiration pressure and normalized center displacement was highly linear in all four finite element models that assigned neo-Hookean material properties. LV: left ventricle, RV: right ventricle, c: control, PTA: persistent truncus arteriosus mutant. These results were obtained from models with the properties given in Table 3. (B) Using linear elastic properties for the ventricles resulted in a curved load-deflection curve (RV-PTA-Linear). Disregarding the porosity of the trabecular layer and modeling it as solid with the same properties as the compact layer produced a linear load-deflection curve but at stiffer values.

With a neo-Hookean stress-strain relationship, the calculated model load-deflection curves were highly linear (Fig 6A), with R^2^ values averaging 0.9994, while in contrast, load-deflection results using linear elastic material properties were nonlinear, concave upward with stiffening at larger strains (Fig 6B). With the optimal neo-Hookean relationship, i.e., that producing the best match of the finite element model results to the experimental results, the low-strain elastic modulus *E* in the mutant hearts increased by a factor of 2.1 in the LV and 3.9 in the RV over the corresponding controls (Table 3). These increases are similar to the increases in experimental SED, 1.7 in LV and 3.7 in RV.

**Table 3:** Material parameters for best fit of the finite element model to experimental results.

	E, small-strain tangent modulus (kPa)	Lame parameter μ (kPa)	Bulk modulus κ (kPa)	Experimental strain energy density (kJ/m^3^)
Left Ventricle—control	5.78	1.94	96.3	.416
Left Ventricle—mutant	12.3	4.13	205.0	.701
Right Ventricle—control	2.42	0.81	40.3	.177
Right Ventricle—mutant	9.48	3.18	158.0	.651

Parameters determined for a neo-Hookean stress-strain relationship. Poisson’s ratio was 0.49 in all cases. The last column contains the experimental strain energy density at a normalized aspirated length of 0.4 for reference.

**Fig 7:**
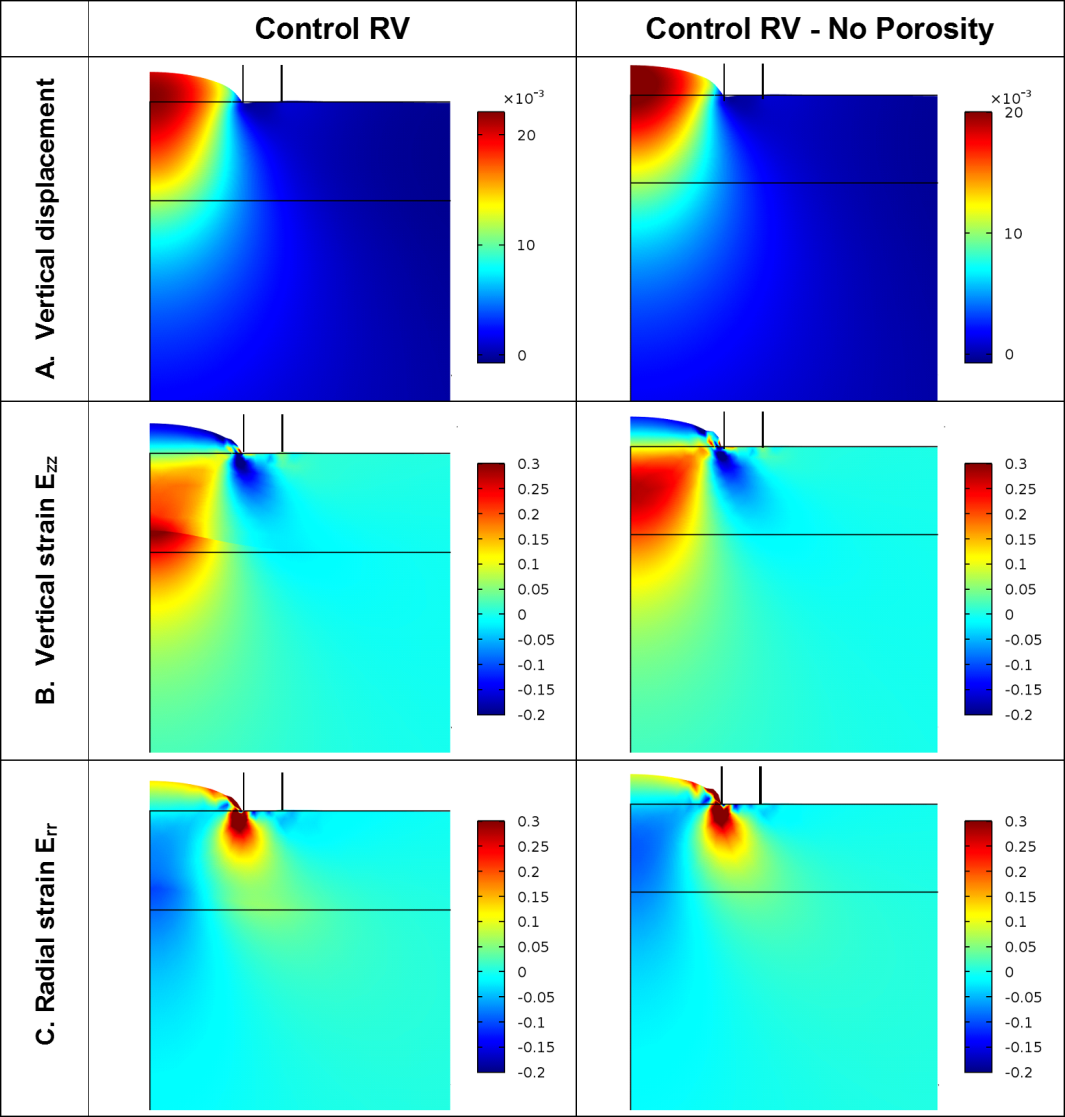
An 0.2 mm x 0.2 mm area of the model showing displacement, vertical Green’s strain E_zz_, and radial Green’s strain *E*_rr_ for a pressure causing a normalized aspirated length of 0.4. A neo-Hookean stress-strain relationship with parameters shown in Table 3 was used in these models. The black outline shows the undeformed geometry, with the horizontal line through the material designating the boundary of the compact and trabecular regions. The pipette outer wall is shown sketched above the top surface of the material. (A) Vertical displacement is smoothly varying and dissipates quickly below the compact-trabecular junction. (B) Vertical strain is negative at the top of the aspirated bubble and has a positive peak at the trabecular-compact junction. (C) Radial strain is positive at the top of the bubble with a negative peak at the junction. Results from the model without trabecular porosity have similar patterns but without the discontinuity at the compact-trabecular junction.

Contour plots of vertical displacement from models using the optimal material parameters showed that the material in the bubble was formed from tissue of the compact layer, although tissue in the trabecular region was also displaced upward by the applied pressure (Fig 7A). At the top of the material bubble, the vertical Green’s strain *E*_*zz*_ was negative, while the radial and circumferential strains *E*_*rr*_ and *E*_*θθ*_ were positive and approximately equal (Fig 7B and 7C). At an aspiration pressure producing $\bar{L}$ = 0.4 in the control RV model, for example, the strains at the top of the bubble were *E*_*zz*_ = -0.156 and *E*_*rr*_ and *E*_*θθ*_ = 0.122. At $\bar{L}$ = 0.2, these strains were *E*_*zz*_ = -0.057 and *E*_*rr*_ and *E*_*θθ*_ = 0.038.

Vertical strain peaked under the center of the pipette at the compact-trabecular junction. Strains decayed quickly with depth in the tissue: to 25% of surface value at an average depth of 0.015 mm (3r_i_) and 10% of surface value at a depth of 0.22 mm (4-5r_i_) (Fig 7B). In the radial direction, strains decayed to 10% of the midline value by 0.17 mm (2r_o_) from the centerline (Fig 7C). The model predicted high stresses and strains at the pipette edge where the tissue bends around the pipette inner corner, although this edge condition is likely not well modeled and, based on St. Venant’s principle, is unimportant to the behavior of the tissue aspirated into the pipette.

The presence of the softer trabecular region under the compact layer influenced the height of the bubble. In analysis of models with identical material properties but no trabecular porosity, higher aspiration pressures were necessary to reach the same aspiration height as that of corresponding models that did not contain the softer trabecular layer (Fig 6B). Also, the displacement and strain fields dissipated faster and the peak in strain at the compact-trabecular junction was absent (Fig 7).

## Discussion

This study used a novel method to measure passive ventricular and conotruncal wall stiffness in embryonic hearts of controls and mutants with developing PTA. We found greatly increased left and right ventricular stiffness in mutant hearts that cannot be attributed to wall thickness or trabecular density. Based on a strain energy measure, stiffness increased 2-fold in the LV and 4-fold in the RV in mutant hearts. Based on the mathematical model, elastic moduli increased by similar multiples of 2.1 in the LV and 3.9 in the RV.

The strain energy density (SED) measure, equal to the area under the experimental pressure versus normalized deflection curve, is physically meaningful and allows direct comparison of fundamental behavior from the PA testing. A similar metric was proposed to compare PA measurements from day 5–8 chick AV cushions and compared well with uniaxial testing in determination of material properties [34]. The use of SED eliminated problems inherent in characterizing hyperelastic materials: the large number of possible mathematical relationships, the common situation of more parameters than can be uniquely determined experimentally, and difficulties comparing parameters between material models. Most biological tissue has a nonlinear, hyperelastic stress-strain relationship, with an approximately linear region at low strains. Because of this, we chose two strain regions for calculation of SED: $\bar{L}$ = 0–0.2 with maximum calculated surface strains 0.04–0.06, and $\bar{L}$ = 0–0.4 with maximum calculated surface strains 0.12–0.16. SED increased in both ranges in both ventricles, but the increase was relatively smaller in the larger strain range, which indicates slightly differing shapes of the nonlinear stress-strain region of the tissue.

When interpreting the increased stiffness of the LV and RV in mutants as a reflection of altered flow, pressure, and other biomechanical inputs, it is important to consider the lineage, genetic, and morphogenic features of the RV compared to the LV in *Fgf8;IslCre* mutants. LV progenitors are derived from *Fgf8*-expressing mesoderm in the primitive streak and early cardiac crescent [35], the first heart field. However, *Isl1Cre* is not active at this early stage [21,22] and thus, LV progenitors in *Fgf8;IslCre* mutants have an intact *Fgf8* locus and are recruited normally to the heart tube. In contrast, beginning at approximately ED 8.5, RV precursors are deployed from the second heart field, the pharyngeal mesoderm caudal to the pharynx, into the heart tube where they develop cellular and molecular features of RV myocardium. Autocrine *Fgf8* signaling is required for normal proliferation, survival, and function of the RV progenitors prior to their differentiation and deployment to the heart [22,36]. In early *Fgf8;IslCre* mutants, the embryonic RV is smaller than normal [22], although it rapidly recovers such that at the time of this study (ED12.5), it is 75% of the size of control RV and normal size by birth [22]. Considering the differences in the genetics and lineage of the RV and LV, we attribute the increased passive tissue stiffness of the LV to altered hemodynamics and aberrant function of the genetic-morphogenetic-biomechanical feedback loop. Because the RV derives from genetically abnormal precursors, increased RV passive stiffness in the mutants is likely a result of the primary genetic defect (which causes an abnormal signaling environment during RV precursor proliferation and deployment) combined with abnormal hemodynamics.

OFT progenitors, like those of the RV, derive from the second heart field and begin to accrue to the heart shortly after ED 8.5 [37–40]. Unlike the recovery seen in the size of the mutant RV, the mutant OFT remains structurally and functionally abnormal: OFT myocardial cells are not properly differentiated and fail to secrete the signaling molecules required for neural crest and endothelial cells to invade the cushions to septate the OFT [22,36]. The OFT remains short and does not remodel or rotate normally. All of these events result in PTA and perinatal death. It is notable that in the normal heart, the OFT has the highest passive stiffness of the three tissues tested and this is not further increased in the mutants, despite the markedly different morphology and composition of the OFT cushions (Fig 4 and [22,39]).

Determining mechanical material properties of any structural material or biological tissue involves applying a known loading, measuring strain, calculating stress with a mathematical model based on physical principles, and implementing a mathematical representation of the stress-strain relationship. One of the simplest examples is the classical uniaxial tension test, where a rectangular strip of engineering material with constant cross-sectional geometry and isotropic elastic material properties is clamped and pulled from the ends. In contrast, the small size (less than 1 mm lateral dimension for LV, Fig 1), irregular geometry, and fragility of the ED12.5 mouse heart present numerous challenges to experimental measurement of material properties. The PA technique used in this study on excised hearts has many advantages over techniques used in chick and later-stage mouse. Dependable *in vivo* measurements are virtually impossible. However, the ventricles and OFT remain intact in PA, whereas extracting strips to secure to a loading fixture would be more destructive. Another method of applying load, sealing the ventricles and creating internal pressurization, requires a means of applying and measuring inflation pressure, measuring surface strain, and modeling the geometry of the entire LV and RV, and would likely omit the OFT. Atomic force microscopy (AFM), or the related techniques of nanoindentation and microindentation, are not as well suited for soft biological tissues and generally involve very small tip sizes, down to tens of nanometers for AFM, with indentations on the order of microns. This may limit the sampling to only the epicardium. *In vivo* strain and strain rate have been estimated through variation of wall thickness in serial images from optical coherence tomography (OCT) in the tubular heart of the 3-day (HH18) chick embryo [41]. However, the chick embryo presents a much more accessible system than the mouse embryo, and strain alone cannot be related to material properties without simultaneous knowledge of load (force or pressure) and geometry, and a stress-strain representation. A limitation to the PA technique is that it does not measure tissue anisotropy, although little could be expected at this stage based on comparison with human development [42]. Although the current PA measurements were done at a consistent, quasistatic rate, this is not a limitation of the technique and a further study could include the effects of different loading rates to assess time-dependent (viscoelastic) material properties.

Ventricular or OFT tissue inhomogeneity may exist, but quantifying spatial variation of material properties was beyond the scope of this project, although measurements at multiple locations on the same ventricle could be done in a future study. The pipette contact locations used in this study were chosen to allow positioning of the pipette perpendicular to the surface, clear video recording of the aspiration process, and maximal repeatability both within groups and between control and mutant groups. The pipette inner diameter of 100 micron was small enough to create localized stress and strain but large enough to cover a continuum of cells: Hirschy et al. [43] measured myocardial cell length of 25.6, width of 9.7, and myofibril length of 18.5 micron in ED12.5 mouse. Using larger pipettes will sample more of the underlying tissue, but is undesirable as more of the surrounding and deep tissue boundary conditions also affect the measurement.

The creation and analysis of the geometrical model in this study was significant in allowing calculation of fundamental mechanical material parameters. To our knowledge, no other measurements exist of passive ventricular wall material properties in ED12.5 mouse. Some comparisons can be made with measurements in later-stage mouse and chick. Jacot et al. [44] used atomic force microscopy with one-micron indentation into the LV free wall of mice at ED13.5 and 16.5. Using a Poisson’s ratio of 0.45 in their Hertzian contact model, they reported 12.0 kPa as an average linear elastic modulus at ED 13.5 and 16.5. Majkut et al. [45], using glass micropipettes of 35–45 micron ID and three different pressures from 0.5 to 20 kPa, found elastic moduli of 0.82 kPa at ED2, 1.1 kPa at ED4, and 1.6 kPa at ED6 in chick, although with very large strains (to more than 2). Tobita et al. [15] combined passive pressure inflation, measurement of epicardial strain, and an assumed geometric model of the ventricles to calculate elastic moduli of 3.43 kPa in RV and 6.13 kPa in LV of HH27 (ED5) chick. Miller et al. [16], using direct uniaxial tension applied to strips cut from the apical region of the chick LV, measured 3.75 kPa at HH27. HH27 or ED5 in chick is an approximate developmental correlate to ED12.5 in mouse [46]. Given that all reported results can be expected to vary with technique and experimental conditions, the calculated results for small-strain tangent elastic modulus of 2.42 kPa for the RV and 5.78 kPa for the LV are in the range of those reported for chick and are slightly lower than those found in older mouse by atomic force microscopy, which uses a very shallow indentation depth.

Analysis of the model produced two other important results. First, it verified that the myocardial tissue is best modeled by a nonlinear stress-strain (constitutive) relationship. This is otherwise slightly counterintuitive, since the load-deflection, or experimental pressure vs aspirated height, relationship is linear. Analysis of models with a linear stress-strain relationship, in contrast, showed a nonlinear pressure-height relationship (Fig 6B). Model analysis also verified that application of aspiration pressure samples tissue below the surface to depths of 0.2 mm and surrounding tissue to a radius of 0.17 mm, based on decay to 10% of surface values of strain and displacement. This is significant for three reasons, demonstrating (1) conditions at the inner endocardial border do not affect the measurement, since this depth represents 44–52% of the way through the trabecular layer in the various geometries, (2) the curvature of the heart does not affect the model, and (3) the experimental measurements sample tissue deep to the surface and thus represent properties of epicardium, compact layer, and trabecular layer. Note that the model results depend on the assumption that the layers are completely bonded and on the simplification of the trabecular modeling.

The development of a whole-mount confocal imaging technique in this study allowed 3D reconstruction of the ventricles and outflow tract of the ED12.5 heart and subsequent calculation of ventricular tissue volumes and trabecular density. The BABB clearing method with tissue autofluorescence has the advantages of being fast, non-aqueous, and requiring no special equipment [47]. Other clearing methods that may preserve fluorescent labelling require long incubation times or complicated procedures (Sca*l*e [48], CLARITY [49]) or limit the time that tissue can be stored in the medium (3DISCO [50], CUBIC [51]). Still others can produce incomplete clearing (SeeDB [52]), or involve more hazardous chemicals (ClearT [53]). The measured trabecular densities in the control hearts of 48.5% in the LV and 40.5% in the RV compare favorably with those measured in the embryonic chick heart: 50% at HH27 (5d) and 46% at HH29 (6d) [16]. Trabecular thickening and compaction are associated with advancing developmental age [54] and also increased pressure [16]. The measured increase in RV trabecular density (39%) and decrease in RV volume (25%) in the mutant hearts (not statistically significant due to wide variability in the mutants) are consistent with advancing RV development and abnormal hemodynamics.

Genetically-based outflow tract defects such as PTA are typically demonstrated through gross structural abnormalities, which in turn often lead to severe functional problems, such as cyanosis, ventricular hypertrophy, pressure anomalies, and eventual heart failure. The structural defects are primarily surgically repaired during the first days or months of life. However, if increased passive ventricular stiffness occurs during development, it may act to worsen the original defect and cause additional defects. If persisting postnatally, increased passive ventricular stiffness is detrimental to cardiac function. Ventricular end-diastolic pressure (EDP) is higher for a given end-diastolic volume (EDV). The passive relaxation phase of diastole is impaired, and the active relaxation phase may also be impaired if the cause of the increased passive stiffness resides partly in the myocardial cells, leading to reduced ventricular filling. Diastolic dysfunction or heart failure with preserved ejection fraction (HFpEF), defined as symptomatic HF with EF ≥ 40%, is now observed in 50% of hospitalizations for congestive HF [55].

In summary, our data indicate that increased tissue passive stiffness of the mutant LV is due to aberrant hemodynamics that changes the output of the genetic-morphogenetic-biomechanical feedback loop. We are in the process of determining the transcriptional and cellular alterations that occur in LV cells during evolution of the PTA defect that lead to its increased tissue stiffness. For example, both titin at a sarcomeric level and collagen in the extracellular matrix have been implicated as main contributors to passive ventricular stiffness [56]. Further studies will examine the molecular changes and progression (or resolution) of passive stiffness through birth. Increased RV passive stiffness likely reflects a combination of the primary genetic defect, the abnormal signaling environment during RV precursor proliferation, deployment and differentiation, and biomechanical forces and signals. We have not yet determined if the mutant RV size recovery occurs by recruiting progenitors normally destined for the OFT (thus contributing to the persistently small mutant OFTs), by increased proliferation of RV myocardial cells after their deployment, or some combination thereof. Notably, despite being the most structurally abnormal, the mutant OFT had normal passive tissue stiffness, further supporting the influence of altered biomechanics and feedback on the abnormal tissue properties of the LV and RV in *Fgf8;Isl1Cre* hearts.
